# Primary therapy of early breast cancer: Egyptian view of 2021 St. Gallen consensus

**DOI:** 10.1186/s43046-022-00156-x

**Published:** 2022-12-26

**Authors:** Hussein Khaled, Yousry Wasef Nada, Kareem Mohamed Ramadan, Shawkat Fekry, Mohamed Samy Seleam, Rabab Gaafar, Mohamed Lotayef

**Affiliations:** 1 Medical Oncology, National Cancer Institute, Cairo University, El-Khalig Square, Cairo, 11796 Egypt; 2 Medical Oncology Department, Maadi Armed Forces Medical Compound, Cairo, Egypt; 3 Radiation Oncology Department, National Cancer Institute, Cairo University, Cairo, Egypt

**Keywords:** Breast cancer, Adjuvant therapy, Egypt

## Abstract

**Purpose:**

The theme of the St. Gallen International Breast Cancer Conference 2021 held virtually for the first time, due to the COVID-19 pandemic, was on tailoring therapies for patients with early breast cancer. A monkey survey that included an Egyptian Panel voted on most of the questions of the original St. Gallen consensus, and some added new questions most relevant to oncology practice in the country, to be able to compare voting results that reflect differences in breast cancer management and decision making.

**Methods:**

The panel included 74 Egyptian scientists from different oncology specialties. Management issues including controversial diagnostic and therapeutic interventions were prepared by a small committee and then projected using the online monkey survey website: https://www.surveymonkey.com. The survey included 130 questions. Results were then analyzed, tabulated, and compared to the voting results of the original St. Gallen consensus.

**Results and conclusions:**

Voting questions and resulting percentages of answers from the Egyptian panel were summarized. There was no consensus between the Egyptian and the original St. Gallen panels on 28/130 statements. They mostly included genetic and pathologic aspects, specifically the routine use of gene signature assays and a few queries involving surgical, radiotherapeutic, and systemic interventions. Probably, available resources and healthcare system differences in Egypt compared to European and the USA were the cause of these differences. This would also be applicable to other low- and low-middle-income healthcare scenarios present in many countries, especially with the present constraints of the COVID-19 pandemic.

## Introduction

The 17th St. Gallen International Breast Cancer Conference that was convened in March 2021 aimed to provide clinical guidance on appropriate management of different aspects of early breast cancer addressing imaging, biomarkers, local management, systemic therapy, survivorship, and different issues related to COVID-19 and to weigh the balance between the benefit of adjuvant treatments and treatment burden including many aspects beyond toxicity, e.g., unaffordable costs of some drugs, or lack of experienced facilities [1].

In view of the current situation of the COVID-19 restrictions, for the third time [2], a panel 74 of Egyptian scientists and clinicians from different specialties headed by Prof. Hussein Khaled arranged for a monkey survey vote on some of the controversial issues of early breast cancer management.

## Methods

Questions for the survey were prepared by a steering committee composed of scientists from different oncologic subspecialties, who have the ability and experience in managing breast cancer cases, and are up to date about recent advances in the field. The chosen questions were adapted from the 17th St. Gallen consensus by Thomssen et al. [3], as well as additional new questions of relevance to the Egyptian situation. The panel openly disclosed any potential conflict of interest. Then, this survey was projected using the online monkey survey website: https://www.surveymonkey.com. The survey included 130 questions. The answers were reported in percentages. Voting percentages were modified after excluding abstained voters.

## Results

Results were then analyzed, tabulated, and compared to the voting results of the original St. Gallen consensus. As expected, voting highlighted both, differences as well as similarities in treatment recommendations for early breast cancer compared to the original Saint Gallen voting and recommendations. Survey data are presented as follows:

### Imaging

When the panel was asked about the value of MRI as a standard procedure, in case of patients with human epidermal receptor 2 (HER2)-positive or triple-negative breast cancer (TNBC) planned to receive neoadjuvant therapy, 76% agreed that MRI being a standard modality to evaluate patients who might be chosen for breast conservation. However, this percentage dropped to 68% in case of estrogen receptor (ER)-positive disease.

In case of the presence of microcalcifications detected by preoperative mammogram, 67% of the panel agreed to perform postoperative mammography after breast-conserving surgery (BCS).

### Genetic and pathologic aspects

Ninety-six percent of the panel preferred to test the genetic panels for hereditary breast cancer in women if they have > 10% risk for hereditary mutation, in algorithms based on family history, age at diagnosis, and tumor subtype [4]. About two thirds of them (67%) chose testing for BRCA1, BRCA2, and PALB2 only, while the remaining one third (33%) added ATM, BARD1, CDH1, CHEK2, NBN, PTEN, STK11, RAD51D, and TP53 to the previously mentioned gene panels.

While the refusal to perform a prophylactic mastectomy in patients with PALB2 mutation was clear (73%), the refusal to add tamoxifen or aromatase inhibitors (for postmenopausal women) in the same patients did not have the same clarity (56% refused).

**Fig. 1:**
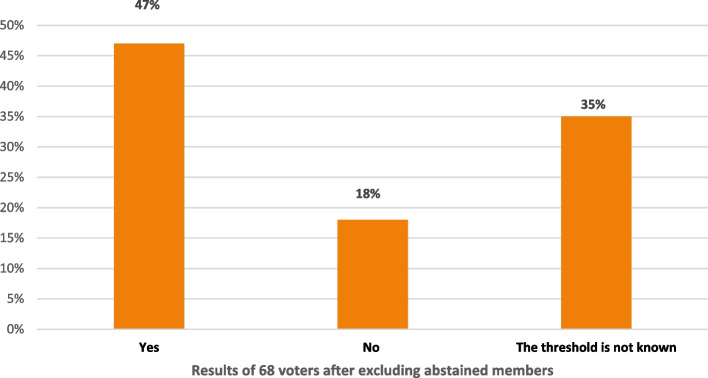
In node-negative ER-positive PR-positive HER2-negative tumors, the Ki-67 threshold that justifies chemotherapy would be a Ki-67 of at least 30%

Although the panel was divided regarding the determination of the Ki-67 threshold that would justify chemotherapy in node-negative, hormonal-positive, and HER2-negative breast cancer as 35% of them decided that the threshold is not known, while only 47% set a threshold level of at least 30% to be the cutoff (Fig. 1), they mostly agreed that in ER-positive, HER2-negative, and T1-2 and N0-1 tumors, a Ki-67 ≤5% would not warrant chemotherapy, while a ki67 ≥30% would justify it (84%). The majority (89%) would test ki-67 in all cases of ER-positive, HER2-negative breast cancer.

When they were asked about Ki-67 testing after neoadjuvant endocrine treatment (NET) to assess its response, 57% agreed, while 43% refused. Sixty percent of the panel confirmed that estimation of prognosis for NET in hormonal-positive, HER2-negative ductal breast cancer could be done using Ki-67 two or more weeks after starting the treatment [5].

**Fig. 2:**
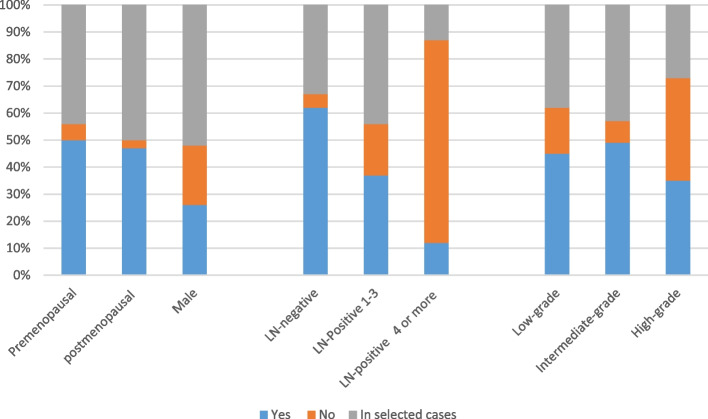
Egyptian panel recommendations for genomic signature testing in ER-positive and HER2-negative early-stage breast cancer

The panelists were asked if the multigene signatures could be used to decide on giving chemotherapy to ER-positive and HER2-negative breast cancer with a 1–3-cm tumor size. The answers varied according to the clinicopathologic factors (Fig. 2):

- Patient’s gender: In male patients, 27% agreed for all males, and 52% agreed only in selected cases. Fifty-seven percent of the panel accepted the statement in all female patients, and 43% accepted only in selected patients.
- Menopausal status: 50% agreed in all premenopausal patients, while 44% agreed but in selected ones. These results were almost the same when the panel was asked about postmenopausal patients, as an agreement in all patients was 47% and 50% agreed in selected patients.
- Nodal status: As the number of positive lymph nodes increases, the percentage of the panel who were accepting to use of multigene signatures to decide on giving chemotherapy decreases. In node-negative tumors, 62% of the panel agreed with all patients and 33% agreed with selected ones. In (1–3 positive lymph nodes) tumors, 37% agreed in all patients and 44% agreed in selected ones. In ≥4 positive lymph node tumors, most of the panel (75%) refused to use the assay.
- Tumor grade: Grade 1 and grade 2 tumors were a point of debate to the voters as 45% and 49% agreed in all patients respectively, also they agreed in selected grade 1 (38%) and 2 (43%) tumors. The area of more debate was the grade 3 tumors as 38% rejected the statement, 35% accepted it in all patients, and 27% reserved the acceptance to selected patients.

Routine testing of additional biomarkers in TNBC was questioned. PDL-1 was chosen to be tested in selected patients (50%), but 36% preferred to test it in all patients, also TLIs were preferred to be tested in selected patients (55%); however, 25% of panelists voted to test it in all patients, while 20% refused testing it at all.

### Ductal carcinoma in situ

In patients with age ≥ 70 years, 87% of the panel advised not to give radiation therapy for ductal carcinoma in situ (DCIS). This was similar to an updated analysis with a median follow-up of 12.6 years of a previous CALGB 9343 study comparing lumpectomy with whole breast radiation or lumpectomy alone, both with tamoxifen for five years in clinical stage 1, ER-positive breast cancer patients 70 years of age or older [6]. Only 57% of the voters would omit radiation therapy in DCIS tumors with single lesions and without necrosis, while 43% would advise to give radiation therapy.

To prevent DCIS recurrence, most of the voters (80%) preferred to use tamoxifen 20 mg daily, this was like the NSABP B-24 trial which found a benefit from tamoxifen for women with DCIS after treatment with BCS and radiotherapy [7]. Aromatase inhibitors (AIs) gained only 11% of the votes.

### Breast surgery

The panelists differed in their opinion regarding the appropriate time and sequence of reconstruction and postmastectomy radiotherapy (PMRT). While 41% preferred delayed reconstruction after radiotherapy, the others split between delayed immediate (expander) (17%), immediate autologous reconstruction (11%), and immediate implant in 1 or 2 stages (10%) with 21% abstaining.

Mastectomy—as a standard procedure for ipsilateral local recurrence after conservative surgery—was the preferred option by 65%, while 32% preferred to do another BCS, then give radiotherapy if the lead time was > 5 years. Factors that would favor a second BCS are low-risk (small, luminal A) tumors (75%) or intermediate risk defined as elapse of 5 years interval since the first diagnosis (72%). Mastectomy was also the standard procedure for ipsilateral local recurrence after BCS if irradiation was not an option (93%). Surgery should not be omitted after neoadjuvant therapy in case of clinical and radiological complete remission (91%). Richter et al. [8] reported that there is good evidence not to avoid surgery as response evaluation cannot be properly confirmed with certainty by using only imaging evaluation.

### Axillary surgery

**Fig. 3:**
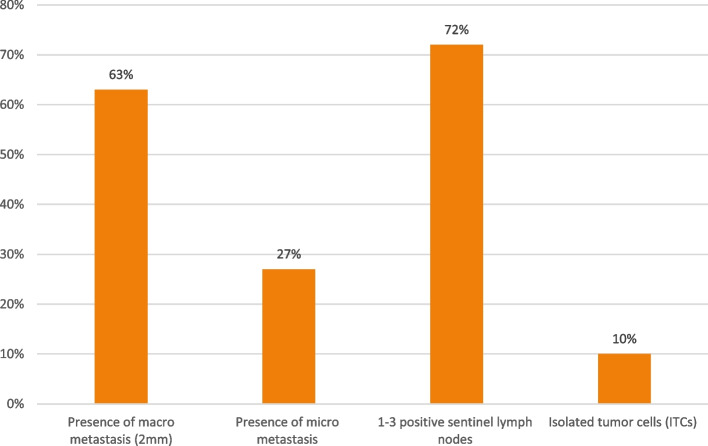
Is axillary dissection required for residual cancer in lymph nodes after standard neoadjuvant chemotherapy?

We asked the panelists in which cases they should perform axillary lymph node dissection (ALND) after neoadjuvant therapy. The answers were (Fig. 3):

- In case of the presence of macrometastasis (63% with).
- In case of the presence of micrometastasis, 57% were influenced by IBCSG 23-01 trial [9], so they were against ALND while 27% of voters agreed to perform dissection.
- In case of the presence of isolated tumor cells (ITCs) (10% with, 43% against).
- In case of the presence of 1–3-positive sentinel lymph nodes (SLN) (72% with, 28% against).

Sixty-three percent of voters agreed that in case of the presence of initial positive lymph node status (pN1) and good clinical response, a biopsied and clipped lymph node at baseline would be sufficient to avoid ALND, if 3/3 SLNs were negative. Thirty-seven percent of the voters could not avoid it for up to negative 3 out of 3 SLNs. ALND was necessary for initial (pN1) tumors if there was no or minor response (97%). Fifty-nine percent of the voters would endorse targeted ALND for favorable biologic subtypes compared to 69% who would recommend it for cN1 tumors clipped nodes that had become cN0.

Removing more than 10 axillary lymph nodes would not add any benefits, for example, in case of more than 5 nodes affected, in view of 60% of the panel. Also, 96% agreed that intercostobrachial nerves should be preserved as a surgical standard.

In case of (cN0), axillary surgery should be avoided if the patient's age was more than 70 (46%). Twenty-five percent of the panel preferred to avoid it only in patients older than 80 years, while 23% believed that age is not a cause of an axillary surgery omission.

Re-sentinel excision with frozen section and ALND were the preferred procedures from the panel’s point of view (58%), in ipsilateral breast recurrence with negative nodes by imaging techniques in previously treated patients with BCS and SLN mapping. However, 27% preferred to do complete axillary node dissection.

### Radiation therapy

**Fig. 4:**
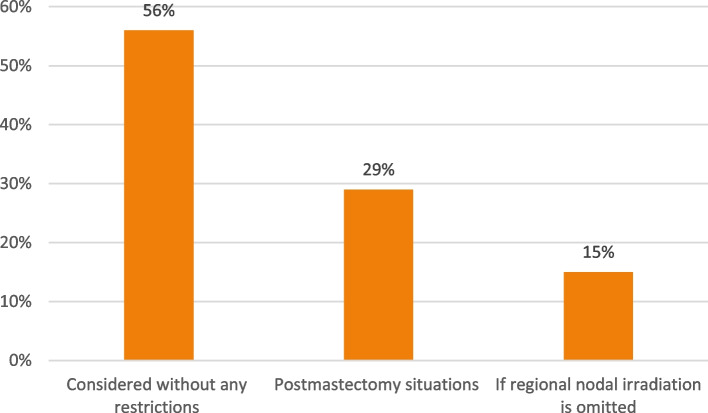
Do you consider hypofractionation?

While hypofractionated radiotherapy for the breast cancer could be considered without any restrictions by 56% of voters, 29% and 15% (Fig. 4) of the panel would consider it for postmastectomy situations or if regional nodal irradiation is omitted, respectively. Standard hypofractionation (15–16 fractions) was preferred by 76% for stage 1 or 2 breast cancer patients who underwent BCS with negative margins. Only 2% recommended ultra-short radiotherapy (5 fractions) for the same previous situation, and 22% recommended giving either of hypofractionation or ultra-short radiotherapy.

The panel was not sure when to recommend partial breast irradiation, as 77% abstained from voting, while 9% would keep it for patients under the age of 40.

Sixty-one percent of panelists did not recommend RNI for patients with triple-negative or HER2-positive breast cancer with pathological complete response (pCR) after neoadjuvant treatment. Seventy-two percent consider it mandatory for stage 2 and (cN1) triple-negative or HER2-positive breast cancer with pCR, while 28% believed that it is mandatory only in stage 3. RNI is necessary for patients with PMRT in case of TNBC (90%), while in luminal A tumors, 33% recommended it in comparison to 67% who considered it unnecessary.

Genomic signatures including Oncotype DX®, MammaPrint®, PROSIGNA®… etc. would not be used to help decide RNI (87%), PMRT (83%), or irradiation omission (80%).

Boost irradiation of the excision site, in case of invasive duct carcinoma (IDC), could be endorsed for high-risk group only (young age, grade 3 tumors, extensive intraductal component (EIC)-positive tumors, TNBC, or HER2-positive tumors) was endorsed by 57% of the panel. This is compatible with data from the START trials [10–13]; although, 43% would consider it for all patients underwent BCS. In DCIS, boost irradiation should not be endorsed in all cases (74%) but was endorsed only in high-risk situations (presence of necrosis, close margins, or large tumor size) by 92%. Also, in patients older than 50 years, 65% would consider giving boost irradiation.

After BCS, omission of radiotherapy is not indicated in more than 70-year-old female patients (62%), node-positive disease (70%), or tumors more than 2.5 cm (70%). We asked them if they might consider radiation omission after BCS in women with less than 2.5 cm tumor size in case of low grade or low genomic score tumors, 52% refused the omission; but, 48% accepted it.

Performing axillary radiation instead of ALND was the subject of some questions to the panel. The answers varied according to the clinical situation:

- Initially cN0, in patients without macroscopic nodal involvement: 57% and 54% accepted in case of (1–3 SLN) with micrometastasis or with ITCs, respectively; although, 43% and 45% refused in the same cases respectively.
- HER2-positive tumors and available TDM-1 therapy: 75% refused, while 25% accepted.
- ER-positive tumors and available endocrine therapy: 68% refused, while 32% accepted.
- TNBC and available capecitabine therapy: 85% refused, while 15% accepted.

### Neoadjuvant therapy

Classically, pCR has been considered as a surrogate endpoint for drug approval in for early stages of breast cancer. Asked about this statement, 59% of the panel voted in favor of this approach if the regimens given achieved a great improvement in pCR rates, i.e., 50% higher than the control. The remaining 41% of panelists suggested that neoadjuvant pCR rates as encouraging, but only improvements in event-free survival (EFS) and overall survival (OS) rates are needed to define “standard” regimens. While 43% of panelists recommended giving neoadjuvant treatment to all patients based only on the initial diagnostic biopsy, 57% of panelists did not agree that this is an appropriate recommendation.

For postmenopausal patients having low-grade and/or low-risk genomic signature disease, 98% did not agree with giving chemotherapy for these patients. In addition, 70% of the votes were in favor of asking for genomic signature assays on core biopsies to decide on giving neoadjuvant chemotherapy versus endocrine therapy to patients with ER-positive breast cancers.

In a case of women with HER2-positive, node-negative breast cancer, 66% of the panel did not favor anthracyclines in patients receiving taxane-based chemotherapy and anti-HER2 antibodies. However, in the case of node-positive cancer, 88% of them would give anthracyclines. Furthermore, in the presence of positive axillary lymph nodes on clinical examination, 49% chose anthracycline-containing regimen, while 51% favored giving platinum- and pertuzumab-containing treatment.

In stages II and III with node-negative axilla, and HER2-positive disease, 60% of the panel agreed to give anthracyclines and pertuzumab in addition to taxane and trastuzumab, while 40% would add pertuzumab and platinum to taxane and trastuzumab.

When treating triple-negative disease, 51% would not add an immune checkpoint inhibitor to the neoadjuvant chemotherapy treatment. Sixty-five percent believed that PD1/PDL1 testing is actually needed to be done to recommend the use of immune checkpoint inhibitors in the neoadjuvant therapy.

### Postneoadjuvant treatment

Eighty-seven percent of the voters agreed that for HER2-positive breast cancer with pCR after neoadjuvant therapy, the baseline stage and tumor subtype still affect patients’ prognosis.

In patient with cN+ HER2-positive breast cancer when pCR is achieved, 52% of the voters would give trastuzumab and pertuzumab adjuvant treatment of choice regardless of baseline stage, and 32% would favor trastuzumab and pertuzumab but only in baseline stage 3, while 16% would give only trastuzumab.

When pCR was achieved with trastuzumab and pertuzumab in patients presenting with cN0, HER2-positive cancer, 67% would favor giving trastuzumab alone as adjuvant treatment, and 21% would agree to consider pertuzumab and trastuzumab if patients had baseline stage 1 or 2 and 12% would give both agents with baseline stage 2 only.

Eighty percent of the voters would offer trastuzumab-emtansine to all patients with residual disease, while the panel was equally split, whether they would offer trastuzumab emtansine also to anti-HER2 treatment in patients with excellent clinical response and <5 mm residual disease.

If pCR is achieved in TNBC, the panel did not agree by a clear majority (81%) to give immune checkpoint inhibitors in the adjuvant setting while a strong majority (97%) would favor adjuvant capecitabine to women having residual disease after neoadjuvant treatment.

For patients with ER-positive disease after neoadjuvant endocrine, the panelists did not agree (68%) to offer adjuvant chemotherapy if they had excellent clinical response short of pCR and node-negative residual cancer, but if there were 4 or more residual lymph nodes, 88% of them would give chemotherapy. If there were any residual positive lymph nodes, 70% would offer adjuvant chemotherapy. If residual tumor size was >5 cm, 87% would give adjuvant chemotherapy. In case of the presence of baseline high-grade tumor and/or intermediate-range genomic signature, 83% would offer adjuvant chemotherapy.

### Adjuvant endocrine therapy

To recommend endocrine adjuvant treatment, for hormone receptor-positive patients, the panel was asked to choose between ER threshold level ≥1 vs. ≥10% tested by IHC; the panel was equally split 50/50, while 97% of votes recommended endocrine treatment with any tumor size that include microinvasive disease for patients having luminal A and B like lesions.

**Fig. 5:**
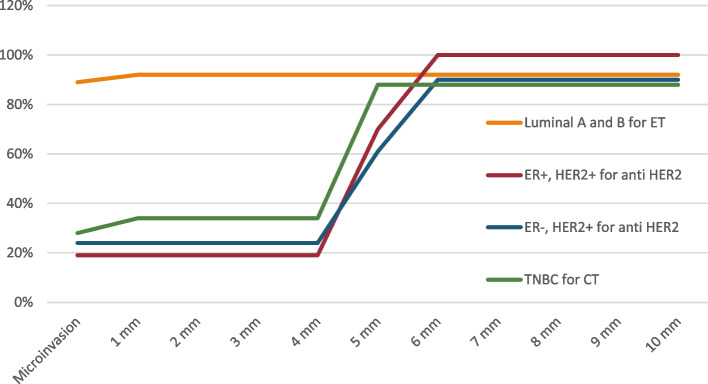
Egyptian panel recommendation of size threshold for initiating systemic therapy by tumor type and treatment

In cases having ER-positive, HER2-positive disease, 51% of the panelists agreed to give anti-HER2 therapy in case of tumor size equal or more than 5 mm or 6 mm (30% of votes), but 19% suggest giving such treatment in smaller lesions. The consensus was less clear to give anti-Her2 therapy to patients having negative ER and positive HER2 disease; votes were 41% for tumor size 5 mm, 32% for 6 mm, and 27% for tumors less than 5 mm to consider anti-HER2 therapy. Results are shown in Fig. 5.

### Endocrine adjuvant treatment in premenopausal cases

**Fig. 6:**
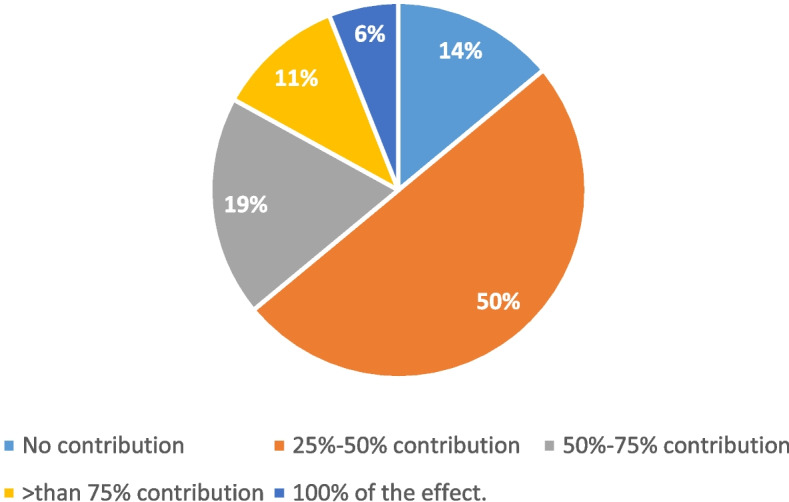
The contribution of chemotherapy-induced ovarian function suppression (OFS) to the total effect of chemotherapy in premenopausal, ER-positive early stage with favorable biological features

In premenopausal patients with ER-positive early-stage disease with favorable biological features, 53% of the panel considered the contribution of chemotherapy-induced ovarian function suppression (OFS) to the total effect of chemotherapy to be at least 25–50% (Fig. 6). In those patients, and to evaluate the effect of OFS, 77% of the voters would use routine monitoring of estradiol levels.

In case of premenopausal with ER-positive, HER2-negative lesions with features necessitating adjuvant chemotherapy, 95% would consider adding OFS, if those patients remain premenopausal after finishing chemotherapy treatment [14].

**Fig. 7:**
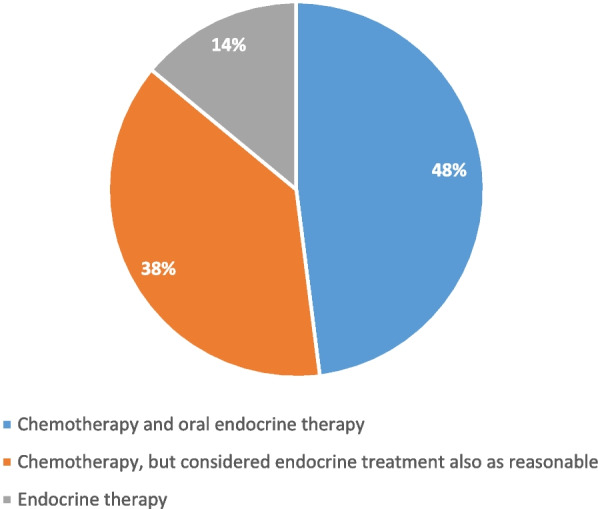
Panel recommendation for premenopausal women with 1–3 positive lymph nodes and recurrence score <25 or other low-range genomic signature

If premenopausal women are characterized as having a disease node-negativity and low-risk genomic signature (e.g., recurrence scores 16–25), most of the panelists (75%) would give only OFS with tamoxifen or AIs, while only 25% voted to add chemotherapy. In low-risk patients with 1–3 positive lymph nodes and low recurrence score genomic signature, 48% would add chemotherapy to the endocrine treatment, 38% favored adding chemotherapy to endocrine treatment but at the same time would consider endocrine therapy alone without chemotherapy as a reasonable treatment, while only 14% favored endocrine therapy alone (Fig. 7).

In stage II disease, 80% of the panel agreed to offer OFS, and this percentage increased to 91% in younger women < 40 years of age. Also, 86% voted to consider OFS only independent from a prior adjuvant chemotherapy, if the women had high-risk features such as age less than 40 years, positive lymph node, high Ki-67, luminal B-like, intermediate, or higher risk genomic signature assays. A lower percentage of the panel (14%) recommended OFS to all patients.

### Endocrine therapy extension beyond 5 years in premenopausal cases

In patients with luminal A-like tumors and positive lymph node disease, 99% of the voters agreed to consider a prolonged endocrine therapy; 34% considered additional 2–3 years, and 65% voted for a total of 10 years. After giving 5 years of OFS and tamoxifen, 68% of the panel recommended OFS (if still premenopausal) together with AIs and 32% agreed to give only tamoxifen for 5 years. This applied to cases of high-risk patients.

### Endocrine adjuvant treatment in postmenopausal cases

A majority of the panel (94%) voted that in case of stage ≤ II, positive ER, negative HER2 disease with low-risk signature assays (e.g., recurrence scores ≤25), patients should not receive chemotherapy. For higher anatomical stages (pT3, N1, or >3 infiltrated lymph nodes), 97% decided to give chemotherapy, and only 3% would withhold it.

For postmenopausal cases having high anatomical stage (e.g., stage III), positive ER, negative HER2 breast cancer, voters agreed by 97% that for patients having ≥10 infiltrated lymph nodes (with very high stages), regardless of biomarkers (95%), even with recurrence score <25 (88%) to give combined chemotherapy and endocrine therapy. However, for some situations, e.g., grade 1, low level of Ki-67, recurrence score <11, or lobular cancer, 91% rejected the idea of giving chemotherapy as well.

### Adjuvant chemotherapy

In patients with positive ER, stage I disease, and negative lymph nodes who are assigned to receive adjuvant chemotherapy, 89% chose to give shorter combinations, such as EC or TC. However, 10% of the panelists voted for a standard dose of the combination of anthracycline/cyclophosphamide /taxane.

With higher-stage disease or higher tumor burden, 66% of panelists recommended a standard dose anthracycline/cyclophosphamide/taxane combination, while 29% voted for the dose-dense combination.

In TNBC, the optimal tumor size to start adjuvant or neoadjuvant chemotherapy was 5mm or more by 62% of panelists, while 32% recommended it with microinvasive disease.

### Adjuvant therapy for positive HER-2 breast cancer

Women with negative lymph node, and positive HER2 disease are not eligible to receive adjuvant pertuzumab plus trastuzumab in view of 78% of the panel. Fifty- two percent of the panel agreed to offer an adjuvant neratinib after (neo) adjuvant trastuzumab/pertuzumab and/or trastuzumab emtansine-based therapy.

For patients having stage I, and positive HER2 disease, 62% did not agree to routinely use trastuzumab-emtansine instead of a combination of paclitaxel/trastuzumab; however, 36% considered justifying the use of T-DM1 in certain situations.

### New drugs

#### Abemaciclib

When the panel was asked if abemaciclib should be recommended in EBC with positive ER and negative HER2 cases having >3 positive nodes. The Panel did not agree (68%).

In other possible situations as for women having positive 1–3 nodes or other unfavorable prognostic factors like grade III, T3 tumor size, or high level of Ki-67, only 52% of the panel agreed to recommend the application of abemaciclib while 39% refused. The panel did not agree (77%) to consider Ki-67 level evaluation to indicate anti-CDK4/6 therapy.

#### Use of immune checkpoint inhibitors in triple-negative breast cancer

Seventy-four percent of panelists did not agree to give women having either stage II or also stage III triple-negative breast cancer (who have not been treated by neoadjuvant but receiving adjuvant chemotherapy) anti-PD1/PDL1 therapy.

#### Use of PARP inhibitor in triple-negative breast cancer

The panel did not agree (74%) to recommend olaparib in case of women having stage II or III triple-negative breast cancer, who have not been treated by neoadjuvant, but receiving adjuvant chemotherapy. A summary of the voting results on the role of systemic therapy in the management of early breast cancer is shown in Tables 1 and 2.

**Table 1:**
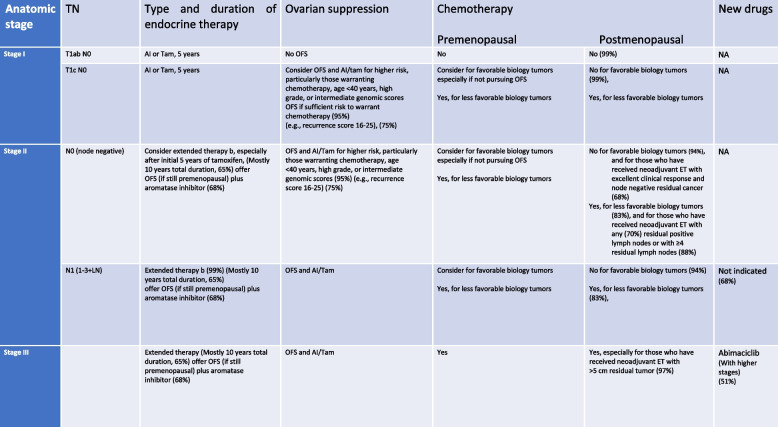
Systemic therapy for ER + HER2-negative breast cancers

**Table 2:**
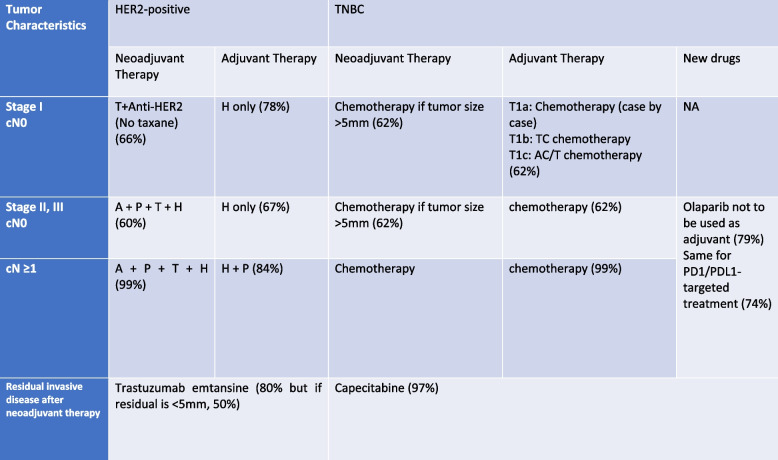
Systematic therapy for HER2-positive or triple-negative breast cancer (voting results)

### Survivorship

Survivorship is now recognized as a phase of cancer that requires ongoing specialized care. Significant genitourinary or sexual health symptoms during the AIs are of concern for some women. Fifty-five percent of the voters answered yes, and 45% would not recommend intravaginal estrogens due to safety concerns.

Chemotherapy-induced alopecia may influence the quality of life and psychological well-being. The panel agreed by a 67% majority on the use of cold caps.

Complementary therapies include a broad range of mind and body practices, natural products, and many lifestyle modifications and are commonly used by breast cancer survivors [15]. The majority of panelists (99%) voted in favor of aerobic exercise, 92% in favor of acupuncture and weight loss, 62%% in favor of encouraging breast cancer patients to take vitamin supplements, and 97% voted in favor of meditation and mindfulness-based stress reduction for breast cancer patients to reduce depressive symptoms.

### Oligometastatic breast cancer

Evidence is emerging that in some patients with “oligometastatic disease,” often defined as five or fewer metastases diagnosed on imaging, aggressive metastasis-directed therapy with surgery, and/or hypo fractionated image-guided radiation therapy improves outcomes and may even be curative [16].

The great majority voters (95%) endorsed for the first-time curative intention for oligometastatic breast cancer (e.g., isolated metastasis in the sternum, isolated metastasis to bone, or single lung nodular), while only 65% endorsed for the first-time curative intention after multiple metastases had responded well to primary systemic therapy.

### COVID-19 and breast cancer

Most of the panelists (90%) voted for endorsing COVID-19 vaccination for all patients before receiving chemotherapy and for all caregivers (99%).

When the panel was asked about prioritizing COVID-19 vaccination, they answered that it should be offered primarily to all women with newly diagnosed breast cancer (49%) and patients with recently completed chemotherapy/radiotherapy (28%). However, vaccination of those with ongoing chemotherapy/radiotherapy was considered less priority (23%).

## Discussion

The main aim of this work was to illuminate both, differences, and similarities in the management concepts of early breast cancer of Egyptian oncologists compared to the original Saint Gallen panel, putting into consideration economic, social, healthcare facilities, and disease patho-biologic factors that are present between breast cancer patients in western countries and most low- or middle-income countries including Egypt.

Out of 130 questions asked to 74 Egyptian scientists, there were major differences in 28 answers (21.5%) between the Egyptian and the St. Gallen panel. These 28 different answers included 11 in biologic and pathologic aspects, 9 in systemic therapy, 3 in surgery, 3 in radiotherapy, and 1 in each of imaging and DCIS (Tables 3 and 4).

**Table 3:** Major differences in voting results between Egyptian and St Gallen Panels (imaging, genetic and pathologic aspects, and local treatment)

Survey Question	Egyptian Panel	St. Gallen Panel
**Imaging**		
Performing post-excision mammography after breast-conserving surgery • no role for the procedure, • Yes, to all patients, • Yes, where initial microcalcifications were identified by mammography, and were treated with breast-conserving surgery,	• Yes, if initial microcalcifications were identified by mammography, and were treated with breast-conserving surgery (67%)	• No role for the procedure (8%) • Yes, to all patients (16%) • Yes, if initial microcalcifications were identified by mammography, and were treated with breast-conserving surgery (18%)
**Genetic and pathologic aspects**		
Testing of genetic panels for hereditary cancer Including: • BRCA1 and 2, ATM, BARD1, BRIP1, CDH1, CHEK2, NBN, PALB2, PTEN, STK11, RAD51D, and TP53 • BRCA1 and 2 and PALB2.	• BRCA1 and 2 and PALB2 (67%).	• BRCA1 and 2, ATM, BARD1, BRIP1, CDH1, CHEK2, NBN, PALB2, PTEN, STK11, RAD51D, and TP53 (67%)
The use of multigene signatures to define chemotherapy needs in ER-positive HER2- negative breast cancer with 1–3 cm (Female patients) • Yes • In selected patients • Never	• Yes (57%)	• In selected patients (72%)
The use of multigene signatures to define chemotherapy needs in ER-positive HER2- negative breast cancer with 1–3 cm (Premenopausal patients) • Yes • In selected patients • Never	• Yes (50%)	• In selected patients (67%)
The use of multigene signatures to define chemotherapy needs in ER-positive and HER2-negative breast cancer with 1–3 cm (Postmenopausal patients) • Yes • In selected patients • Never	• Yes (62%)	• In selected patients (64%)
The use of multigene signatures to define chemotherapy needs in ER-positive and HER2-negative breast cancer with 1–3 cm (Node negative) • Yes • In selected patients • Never	• Yes (62%)	• In selected patients (64%)
The use of multigene signatures to define chemotherapy needs in ER-positive and HER2-negative breast cancer with 1–3 cm (node-positive 1–3)	• Yes (35%) • In selected patients (44%) • Never (21%)	• In selected patients (83%)
The use of multigene signatures to define chemotherapy needs in ER-positive and HER2-negative breast cancer with 1–3 cm (Grade 1)	• Yes (45%) • In selected patients (38%) • Never (17%)	• In selected patients (60%)
The use of multigene signatures to define chemotherapy needs in ER-positive and HER2-negative breast cancer with 1–3 cm (Grade 2)	• Yes (49%) • In selected patients (43%) • Never (8%)	• In selected patients (72%)
The use of multigene signatures to define chemotherapy needs in ER-positive and HER2-negative breast cancer with 1–3 cm (Grade 3)	• Yes (35%) • In selected patients (38%) • Never (27%)	• In selected patients (61%)
In triple-negative tumors, routine testing of additional biomarkers (namely PD-L1 status)	• In selected patients (50%)	• No (93%)
In triple-negative tumors, routine testing of additional biomarkers (namely TLIs)	• In selected patients (55%)	• No (61%)
**Ductal carcinoma in situ**		
Radiotherapy can be omitted for DCIS patients with “unifocal no necrosis” tumors	• Yes (57%)	• No (53%)
**Breast surgery**		
For ipsilateral local recurrence after conservative surgery	Mastectomy is the standard procedure (65%)	Another breast conservation procedure with radiotherapy if the lead time is more than 5 years (63%)
For ipsilateral local recurrence after conservative surgery and if reirradiation is not an option	Mastectomy is the standard procedure (93%)	• Mastectomy is the standard procedure (50%) • Another breast conservation procedure without radiotherapy (50%)
**Axillary surgery**		
After initial positive lymph node status (pN1) and good clinical response, a biopsied and clipped node would be reliable for avoiding ALND when 1 out of 1 sentinel node, or 2 out of 2, or 3 out of 3 were negative, or none of the above	3 out of 3 were negative (63%)	There was no consensus
**Radiotherapy**		
RNI (regional nodal Irradiation) was declined for patients with TNBC or HER2-positive breast cancer with pathologically proven complete response (pCR) after neoadjuvant therapy	No (61%)	Yes (89%)
RNI is considered necessary for patients with PMRT for TNBC	Yes (90%)	No (79%)
Omission of radiotherapy after breast conservation may be indicated in women with tumors <2.5 cm and low-grade or low-genomic score	No (52%)	Yes (88%)

**Table 4:** Major differences in voting results between Egyptian and St Gallen Panels (systemic therapy)

Survey question	Egyptian Panel	St. Gallen Panel
**Neoadjuvant Therapy**		
Should regimens improving pCR rates become standard neoadjuvant therapy	If the regimens achieved a remarkable improvement in pCR rates (50% higher than control) (59%)	Only in case of improvement of event-free survival and overall survival endpoints (83%)
In clinically positive axillary lymph nodes, and HER2-positive tumors, the treatment should contain: • Platinum- and pertuzumab-containing treatment • Anthracyclines	• Platinum- and pertuzumab-containing Treatment (51%)	• Anthracyclines (62%)
In case of stage 2 and 3 clinically node-negative and HER2-positive disease, patients should receive: • Anthracyclines and pertuzumab in addition to taxane and trastuzumab • Pertuzumab and platinum in addition to taxane and trastuzumab	• Anthracyclines and pertuzumab in addition to taxane and trastuzumab (60%)	• Anthracyclines and pertuzumab in addition to taxane and trastuzumab (35%) • Pertuzumab and platinum in addition to taxane and trastuzumab (27%)
Do you see PD1/PDL1 testing affecting the recommendation for the use of immune checkpoint inhibitors in the neoadjuvant therapy	Yes (65%)	No (81%)
**Post neoadjuvant treatment**		
For patients with ER-positive disease after neoadjuvant endocrine treatment and not achieving pCR, should you offer these patients adjuvant chemotherapy if they had an excellent clinical response and node-negative residual cancer	No (68%)	Yes (100%)
**Extension of endocrine therapy in premenopausal patients**		
How to treat high-risk premenopausal patients who had finished 5 years of OFS plus tamoxifen with regards to the type endocrine therapy extension: • Offer OFS (if still premenopausal) plus an aromatase inhibitor • Offer tamoxifen only for 5 years	• Offer OFS (if still premenopausal) plus aromatase inhibitor (68%)	• Offer OFS (if still premenopausal) plus, an aromatase inhibitor (41%) • Offer tamoxifen only for 5 years (45%)
In general, in a case of postmenopausal patients with higher anatomical stages like pT3pN1 or >3 infiltrated lymph nodes, do you recommend chemotherapy	Yes (97%)	No (51%)
**New drugs**		
Do you recommend offering abemaciclib in ER-positive and HER2-negative patients with more than 3 positive axillary lymph nodes	No (69%)	Yes (54%)
In other possible situations as for patients with 1–3-positive lymph nodes or other factors of an unfavorable prognosis such as G3, T3, or high Ki-6, do you recommend the application of abemaciclib	Yes (52%)	No (54%)

As shown by Thomssen et al [3], several important recent research issues in the management of early breast cancer were consented to by the 2021 St. Gallen panel. These issues include endorsement of the possibility of doing another breast-conserving surgery for ipsilateral recurrence, the use of radiotherapy hypofractionation in most of the radiotherapy indications, and the value of including OFS for premenopausal women with luminal breast cancer.

On the other hand, the panel denied the idea of omitting surgery after a good response to neoadjuvant therapy, the importance of pCR for approving new treatments, the use of checkpoint inhibitors for patients with triple-negative stage 2 or 3 disease that have not been treated in the neoadjuvant setting, but receiving adjuvant chemotherapy, and finally adding pertuzumab for node-negative HER2-positive breast cancer.

It is worth mentioning that the Egyptian panel agreed on 4 of above 7 statements. The other 3 statements include their preference in still considering mastectomy as the standard approach after ipsilateral recurrence, probably since most breast cancer patients in Egypt usually present with more advanced local disease than in Europe or the USA. Also, they agreed on the importance of pCR for approving new treatments by a close majority of 59%. Lastly, the Egyptian panel did not recommend offering abemaciclib in ER-positive HER2-negative patients with more than 3 positive axillary lymph nodes but recommended adding anthracyclines and pertuzumab in addition to taxane and trastuzumab in case of stage 2 and 3 clinically node-negative HER2-positive diseases. Based on this survey, possible summary statement guidelines need to be formulated to help in clinical decision-making for Egyptian health authorities and oncology professionals.

## Conclusion

There were clear differences in consensus percentages between the Egyptian and the original St. Gallen panels. Mostly, these differences reflect breast cancer management concepts in Egypt compared to other countries as well as available general healthcare infrastructure as well as oncology management governance, especially with the present COVID-19 pandemic.

## Data Availability

The datasets used and/or analyzed during the current study are available from the corresponding author on reasonable request.

## Abbreviations

HER2: Human epidermal receptor 2
TNBC: Triple-negative breast cancer
ER: Estrogen receptor
BCS: Breast-conserving surgery
DCIS: Ductal carcinoma in situ
AIs: Aromatase inhibitors
PMRT: Postmastectomy radiotherapy
ALND: Axillary lymph node dissection
pCR: Pathological complete response (pCR)
IDC: Invasive duct carcinoma
EIC: Extensive intraductal component
EFS: Event-free survival
OS: Overall survival
OFS: Ovarian function suppression
